# Assessing the Feasibility, Acceptability, and Effectiveness of a Pilot Hepatitis C Screening Program at Food Distribution Sites in Cherokee Nation, Oklahoma

**DOI:** 10.1007/s10900-023-01264-y

**Published:** 2023-08-02

**Authors:** Whitney Essex, Jorge Mera, Ashley Comiford, Amanda Winters, Molly A Feder

**Affiliations:** 1 Department of Infectious Diseases, Cherokee Nation Outpatient Health Center, Cherokee Nation Health Services, 19600 East Ross St, Tahlequah, Ok 74464 USA; 2 Cardea Services, 1809 7th Ave #600, Seattle, WA 98101 USA

**Keywords:** American Indian, Alaska Native, Hepatitis C, Screening, Community Setting

## Abstract

Compared with other racial and ethnic groups in the United States, American Indian and Alaska Native (AI/AN) people experience the highest incidence of acute hepatitis c (HCV). Cherokee Nation Health Services (CNHS) implemented a pilot health screening program from January through May 2019 to assess whether conducting HCV and other preventive health screenings at food distribution sites is a feasible, acceptable, and effective strategy to increase health screening among underserved community members. Data were collected among 340 eligible participants. Most (76%) participants reported being very comfortable receiving health screenings at food distribution sites and that getting screened at food distribution sites is very easy (75.4%). Most (92.1%, n = 313) participants received HCV screening, with 11 (3.5%) individuals testing positive for HCV antibodies. Of the 11 HCV seropositive individuals, six were confirmed to have active HCV infection of which four initiated treatment. Most (55.7%) participants exhibited a body mass index in the obese range, 33.1% exhibited high hemoglobin A1C (> 6.0), 24.5% exhibited high (> 200) cholesterol, 44.6% exhibited high blood pressure ( > = 140/90), and 54.8% did not have a current primary care provider. This project demonstrated that conducting HCV and other health screenings at food distribution sites within Cherokee Nation was an effective strategy to engage AI/AN people in preventive health screenings. Future programs are needed to scale-up preventive health screenings outside of traditional medical facilities as these types of screenings may help to decrease the HCV disparities among AI/AN people.

## Introduction

Hepatitis C (HCV) is one of the most commonly reported notifiable conditions in the United States (U.S.), with approximately 107,300 newly identified chronic HCV cases and 66,700 acute HCV infections in 2020 [1]. Compared with other racial and ethnic groups in the U.S., American Indian and Alaska Native (AI/AN) people are disproportionately impacted by HCV, experiencing the highest rates of acute HCV, at 2.1 cases per 100,000 people in 2020, and deaths due to HCV, with a mortality rate 3.2 times higher than non-Hispanic White people [2, 3]. Studies have shown that people with HCV infection commonly experience HCV-related stigma, and that HCV-related stigma can impact access to and uptake of healthcare [4, 5].

In addition to HCV, AI/AN people experience disparities in other transmissible diseases, including human immunodeficiency virus (HIV) and syphilis, and chronic non-transmissible health conditions, including diabetes, obesity, and chronic kidney disease [6, 7]. For instance, in 2018, AI/AN people were nearly three times more likely to be diagnosed with diabetes and twice as likely to be diagnosed with end stage renal disease than non-Hispanic white adults [8]. In 2018 AI/AN males had an HIV incidence of 16.2 per 100,000 people, compared with 9.6 per 100,000 people in White males, and AI/AN females had an HIV incidence of 3.0 per 100,000 people, compared with 1.7 per 100,000 people in White females [7]. Research has demonstrated that AI/AN people are also less likely to access health care than non-Hispanic white people, including seeing a doctor, visiting emergency departments, and taking needed medications [9].

In response to the rising need for HCV care and treatment among AI/AN people, Cherokee Nation Health Services (CNHS) implemented an HCV Elimination Program in 2015. This program includes universal screening for HCV, expansion of the primary care HCV workforce, and harm reduction interventions. Since the CNHS HCV Elimination Program began, screening rates have increased for those currently receiving healthcare in CNHS [10]. However, a portion of the Cherokee population does not access CNHS and has therefore been missed for HCV screening through the program.

Community-based HCV and other health screenings have frequently been used to reach those who are missed by traditional health clinic or hospital-based screening in the U.S. For instance, health screenings at faith-based institutions and senior centers have been assessed as ways to increase screening for breast and cervical cancer [11], hypertension and cardiometabolic risks [12–14], and diabetes [13, 14] among specific minority groups, including Asian and rural African American women. Several studies have assessed HCV screening programs based in correctional facilities [15–17], homeless shelters [17, 18], and mobile medical clinics [19, 20] and have largely found these settings to be feasible and effective for HCV testing, especially when opt-out testing is implemented [21]. Research has suggested that opt-out testing, which includes notifying individuals that a test will occur unless they decline, can be an effective strategy for reducing stigma around testing and increasing testing rates for stigmatized conditions [22]. Additional locations for HCV testing that have been studied include community health centers [23], senior citizen recreation centers [24], and pharmacies [25].

Few studies have assessed the feasibility or effectiveness of conducting community-based HCV or other health screenings among AI/AN communities or the use of food distribution sites, also known as food pantries, to conduct health screenings. However, existing studies have found that non-clinic-based health screenings are acceptable [26] and useful [27, 28] for identifying disease among AI/AN communities, and that conducting health screenings among non-AI/AN people at food pantries supports identification of people with unmet healthcare needs [29, 30].

In an effort to increase the number of AI/AN people receiving HCV and other important healthcare screenings and detect individuals who could benefit from health services available through CNHS, we implemented a pilot health screening program at two food distribution sites in Cherokee Nation. Food distribution sites are frequented by community members who may be experiencing economic and social hardships, which may prevent them from accessing medical care and staying up to date on important primary health screenings [31].

Through this pilot project, we aimed to assess whether conducting health screenings at food distribution sites is an acceptable and effective strategy to increase HCV and other health screening among underserved community members within Cherokee Nation. Specifically, the project aims were to assess the feasibility and acceptability of conducting health screening at food distribution sites; assess whether conducting health screenings at food distribution sites is an effective strategy for reaching and expanding the population of community members that receive health screening; and identify demographic and clinical characteristics of individuals who frequent food distribution sites, including HCV status. Finally, we aimed to describe the demographic and clinical characteristics of those who were screened and tested positive for HCV.

## Methods

### Setting

Cherokee Nation is the largest federally recognized tribe in the U.S., with a 14-county reservation area in northeastern Oklahoma [32]. The Cherokee Nation Food Distribution Program operates seven food distribution stores within the reservation that provide food to eligible AI/AN people [33]. To be eligible for the Cherokee Nation Food Distribution Program, one household member must be a citizen of a federally recognized tribe, the household must reside within the Cherokee Nation tribal jurisdictional area, and household income must not exceed the U.S. Department of Agriculture’s Food and Nutrition Service income eligibility requirements [33].

This pilot project was conducted at two Cherokee food distribution sites. The first site is located in Tahlequah, a city of 16,463 people, 30.6% of whom are AI/AN [34]. Health screenings at the Tahlequah food distribution site occurred from January 12th through 19th, 2019. The second site is located in Sallisaw, a city of 8,483 people, 20.4% of whom are AI/AN [35]. Health screenings at the Sallisaw food distribution site occurred from May 1st through 15th, 2019.

This study was approved by the Cherokee Nation Institutional Review Board on November 26th, 2018. This study also received approval from the participating food distribution site authorities.

### Planning and Feasibility Assessment

In this pilot project, feasibility was defined as agreement among project staff that the implementation of the health screenings was able to be successfully undertaken [36, 37]. As part of planning procedures, and to assess potential feasibility of health screenings at food distributions sites, project staff conducted pilot observation days to better understand the process of receiving food from the food distribution store, the volume of community members attending the food distribution sites and times of high traffic at participating food distribution sites. These observation days informed the pilot health screening approach, including which days and times would be best to conduct screenings.

Three pilot observation days were conducted during a one-week period in August 2019 at the Tahlequah food distribution site, including a Monday, Thursday and Friday. Observations were conducted during a one to two-hour period between 10:00am and 3:30pm on a given day. A total of 207 individuals were observed entering the Tahlequah food distribution site across the three observation days. Approximately half (50.7%; n = 105) of these individuals were observed on Monday, and the rest were spread across the other two days, 61 (29.5%) individuals on Thursday and 41 (19.8%) individuals on Friday. The middle of the day, around 1:00pm, appeared to be the busiest time. Informed by the pilot observation days, project staff decided to implement screenings in the early afternoon at the two food distribution sites to offer the most individuals the opportunity to receive health screenings without impeding the food distribution site staffs’ workflow.

### Food Distribution Site Health Screenings

#### Eligibility

All unique AI/AN people 18 years and older who visited the food distribution sites during the pilot project period, who were able and willing to provide consent, were eligible to participate in health screenings.

#### Recruitment and Enrollment

To recruit participants for the health screenings, healthcare workers were stationed at check in areas when individuals checked in at the sites to redeem their food vouchers. Healthcare workers greeted and informed people who arrived at the site of the health screening event occurring that day. All interested individuals then proceeded to a table that was set up close to the waiting area to receive information on eligibility, services offered, and to complete consent procedures.

**Fig. 1:**
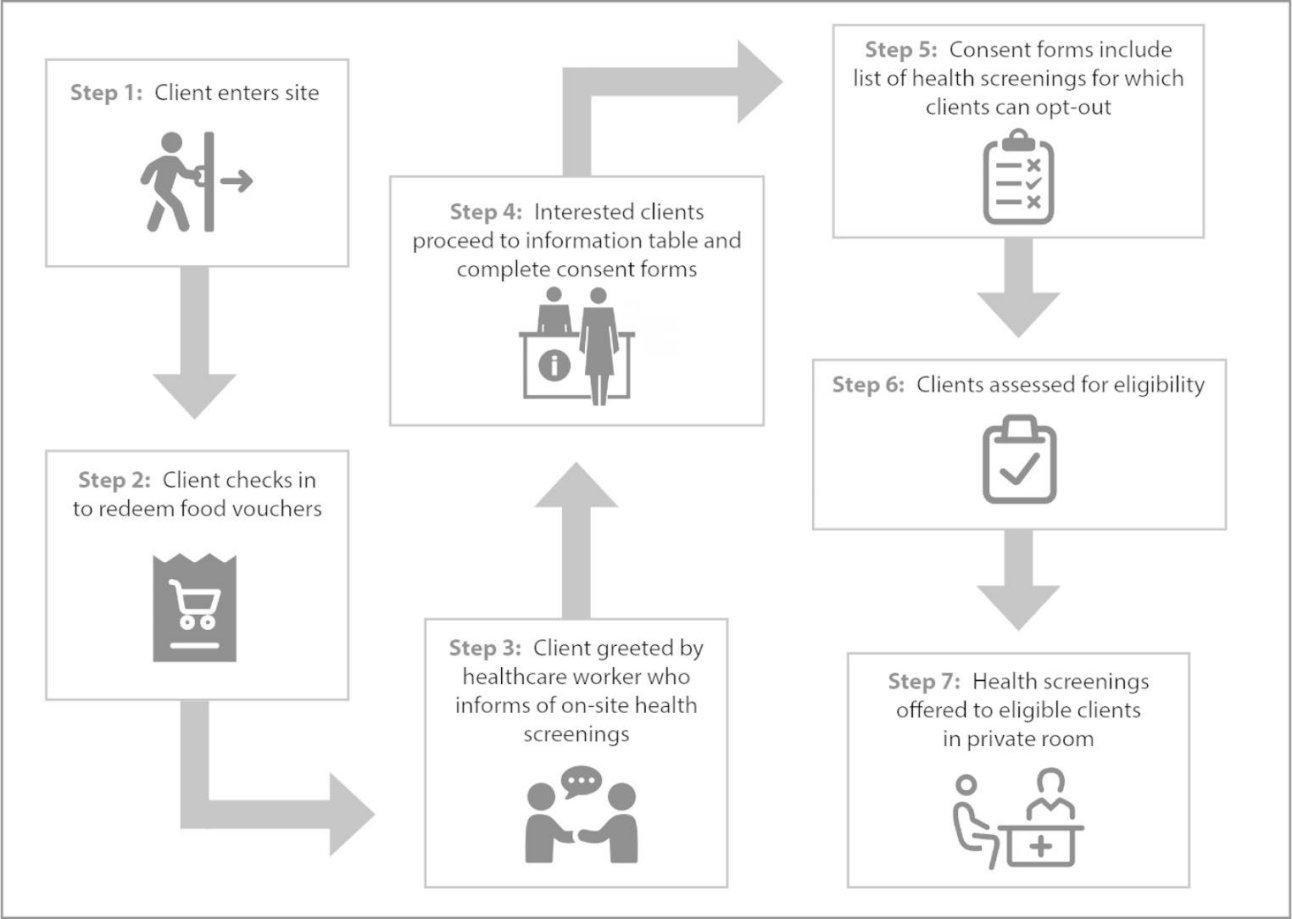
Process for participant recruitment, enrollment, and specimen collection for health screenings at food distribution sites, 2019

During the consent process, eligible individuals received a list of health screening options from which they could opt-out of any screenings they did not wish to receive. The project team decided to implement this testing protocol based on research suggesting that providing patients with the opportunity to opt-out of specific screenings it can be an effective strategy for reducing stigma around testing and increasing testing rates for stigmatized conditions [22]. This list of screenings included screenings for HCV, Human Immunodeficiency Virus (HIV), syphilis, blood pressure, calculated body mass index (BMI), diabetes/pre-diabetes, and lipids profile. Interested individuals were then assessed for eligibility and recruited for participation (Fig. 1). All enrolled participants were offered a $20 gift card for their participation in the health screenings, regardless of which screenings they chose to receive.

#### Data Collection

Clinical staff administering health screenings collected all survey measures for this pilot project using a paper-based intake form. This intake form included questions regarding eligibility for project enrollment. If the individual was eligible to participate in health screenings, the staff member continued a structured interview to complete acceptability and demographic intake form questions. Clinical staff also recorded all screenings received, and later recorded screening results, using the same form. If the participant was enrolled at CNHS and received a health screening result outside of the normal range, clinical staff reviewed participant electronic medical records to assess whether the participant had a recorded history of the health screening result outside of the normal range and if it was being addressed. For people who did not have a history of a screening test being outside of the normal range, or if a screening test outside of the normal range had not been addressed, they were offered a follow-up appointment. If people did not have an existing medical record and were eligible for services, a medical record was created for them.

#### Measures

Acceptability measures included participant-reported comfort receiving health screenings at a food distribution site, using a 4-point Likert scale from very comfortable to very uncomfortable, as well as participant-reported ease receiving health screenings at a food distribution site, using a 4-point Likert scale from very easy to very difficult.

Additional measures included screening date, screening location, current enrollment in CNHS, gender, age, marital status, highest level of education completed, employment status, housing status, health insurance status, lifetime history of homelessness, homelessness in the past six months, lifetime history of incarceration, incarceration in the past six months, frequency of tobacco use, frequency of alcohol use, lifetime history of injection drug use, injection drug use in the past six months, and indicators related to all services received at the pilot screening, including screening for HCV, HIV, syphilis via rapid plasma regain (RPR) test, blood pressure, weight, height, calculated BMI, diabetes via hemoglobin A1C (A1C) and estimated average glucose (eAG), and lipids profile [low-density lipoprotein cholesterol (LDL), high-density lipoprotein cholesterol (HDL), and triglycerides], and the results from these screenings.

#### Specimen Collection

Health screenings occurred in a private room set up specifically for the screenings that day. Phlebotomy was the standard method for conducting laboratory testing. Participants accepting phlebotomy were screened for HIV, HCV, cholesterol level, syphilis, and A1C, or by a combination of these as requested by each participant. Participants that declined phlebotomy were offered HCV rapid testing by finger stick.

Abnormal laboratory findings were communicated to the participant via telephone or face-to-face. If the participant had a primary care provider (PCP) and agreed for this information to be shared with the PCP, the results were forwarded to them for follow-up. If the participant did not have a PCP, the participant was given the option to come to the CNHS Specialty Clinic for an appointment or walk-in visit at their convenience.

#### Data Analysis

Quantitative participant data were analyzed using descriptive statistics including counts, proportions, and graphs. All quantitative analyses were performed using IBM SPSS statistics 19 [38].

## Results

### Health Screening Participant Demographics

Table 1 describes the demographic characteristics of participants. A total of 356 people were screened for eligibility to participate in food distribution site screenings, 340 (95.5%) of whom were eligible to participate. Among the 340 eligible individuals, 184 (54.1%) were screened at the Tahlequah site and 156 (45.9%) were screened at the Sallisaw site. Approximately two-thirds (66.0%) of all participants were female. Compared with other age ranges, the largest proportion of participants (39.8%) were 40 to 59 years of age. About two-fifths (42.8%) of participants reported being single, while 33.0% reported being married. Three-fourths (75.5%) of participants had a high school education or less, while the remaining 24.5% of participants reported a college education. Two-thirds (66.3%) of participants were unemployed (Table 1).

**Table 1:** Demographics among 340 participants who were screened at food distribution sites, 2019

Characteristic	Tahlequah (n = 184)		Sallisaw (n = 156)		Total (N = 340)	
	n	%	n	%	N	%
Gender	182				338	
Female	115	63.2	108	69.2	223	66.0
Male	67	36.8	48	30.8	115	34.0
Age	182		154		336	
20 to 29 years	26	14.3	15	9.7	41	12.2
30 to 39 years	34	18.7	16	10.4	50	14.9
40 to 49 years	39	21.4	31	20.1	70	20.8
50 to 59 years	38	20.9	26	16.9	64	19.0
60 to 69 years	21	11.5	33	21.4	54	16.1
70 years or older	24	13.2	33	21.4	57	17.0
Marital status	183				339	
Single	95	51.9	50	32.1	145	42.8
Married	45	24.6	67	42.9	112	33.0
Divorced	31	16.9	16	10.3	47	13.9
Widowed	12	6.6	23	14.7	35	10.3
Education level	180		147		327	
High school or less	128	71.1	119	81.0	247	75.5
College (Associate level)	28	15.5	21	14.3	49	15.0
College (Bachelor level)	15	8.3	5	3.4	20	6.1
Graduate school	9	5.0	2	1.4	11	3.4
Employment status			151		335	
Not employed	114	61.9	108	71.5	222	66.3
Employed, full time	60	32.6	32	21.2	92	27.5
Employed, part time	10	5.4	11	7.3	21	6.3
Housing status	181		148		329	
Permanent housing	133	73.5	126	85.1	259	78.7
Temporary housing	41	22.7	18	12.2	59	17.9
Homeless	7	3.9	4	2.7	11	3.3
Lifetime history of homelessness			151		335	
No	144	78.3	131	86.8	275	82.1
Yes	40	21.7	20	13.2	60	17.9
Homeless, past 6 months	38		20		58	
No	28	73.7	17	85.0	45	77.6
Yes	10	26.3	3	15.0	13	22.4
Lifetime history of incarceration	183		147		330	
No	154	84.2	119	81.0	273	82.7
Yes	29	15.8	28	19.0	57	17.3
Incarceration, past 6 months	30		26		56	
No	26	86.7	24	92.3	50	89.3
Yes	4	13.3	2	7.7	6	10.7
Insurance status	182		149		331	
Insured	102	56.0	77	51.7	179	54.1
No insurance	80	44.0	72	48.3	152	45.9
Enrolled at CNHS			153		337	
Yes	156	84.8	143	93.5	299	88.7
No	16	8.7	2	1.3	18	5.3
Unsure	12	6.5	8	5.2	20	5.9
Primary care provider	179		151		330	
No	107	59.8	74	49.0	181	54.8
Yes	72	40.2	77	51.0	149	45.2

Fifty-nine (17.9%) participants reported living in temporary housing, 11 (3.3%) reported being currently homeless, and 60 (17.9%) reported experiencing homelessness in their lifetime. Among those who reported experiencing homelessness in their lifetime, 22.4% reported experiencing homelessness within the past six months. Fifty-seven (17.3%) participants reported being incarcerated in their lifetime and six (10.7%) of these individuals reported incarceration within the last six months (Table 1).

### Health Care Status

Among respondents, slightly less than half of participants (45.9%) reported being uninsured. Most respondents (88.7%, n = 299) reported currently being enrolled at CNHS at the time of their health screening and 11.2% (n = 38) were either not enrolled or were unsure if they were enrolled at CNHS. Fewer than half of pilot participants (45.2%) reported having a current PCP (Table 1).

### Substance Use

Across pilot health screening participants, approximately two-fifths (40.1%) reported using tobacco daily and approximately 12% reported using alcohol either weekly or daily. Twenty-six (7.8%) participants reported injecting drugs in their lifetime, six of whom (26.1%) reported that they had injected drugs in the past six months (Table 2).

**Table 2:** Substance use among 340 participants screened at food distribution sites, 2019

Characteristic	Tahlequah (n = 184)		Sallisaw (n = 156)		Total (N = 340)	
	n	%	n	%	N	%
Tobacco use	179		150		329	
Never	88	49.2	75	50.0	163	49.5
Monthly or less than 1 time/month	12	6.7	6	4.0	18	5.5
Weekly	10	5.6	6	4.0	16	4.9
Daily	69	38.5	63	42.0	132	40.1
Alcohol use	183		149		332	
Never	109	59.6	106	71.1	215	64.8
Monthly or less than 1 time/month	49	26.8	28	18.8	77	23.2
Weekly	19	10.4	10	6.7	29	8.7
Daily	6	3.3	5	3.4	11	3.3
Lifetime history of injecting drugs			151		335	
No	171	92.9	138	91.4	309	92.2
Yes	13	7.1	13	8.6	26	7.8
Injected drugs, past 6 months	12		11		23	
No	7	58.3	10	90.9	17	73.9
Yes	5	41.7	1	9.1	6	26.1

### Acceptability

Most pilot participants reported being very (76%) or somewhat (21.6%) comfortable receiving health screenings at food distribution sites, with a higher proportion of “very comfortable” participants in Sallisaw (86%) compared with Tahlequah (67.8%). Most participants reported that getting screened at food distribution sites is easy, with three-fourths (75.4%) reporting that getting screened at food distribution sites is very easy (Table 3).

**Table 3:** Acceptability of health screening at food distribution sites among 340 participants, 2019

Characteristic	Tahlequah (n = 184)		Sallisaw (n = 156)		Total (N = 340)	
	n	%	n	%	N	%
Comfort getting screened at food distribution site	183		150		333	
Very comfortable	124	67.8	129	86.0	253	76.0
Somewhat comfortable	54	29.5	18	12.0	72	21.6
Somewhat uncomfortable	5	2.7	2	1.3	7	2.1
Very uncomfortable	0	0	1	0.7	1	0.3
Ease of getting screened at food distribution site	178		147		325	
Very easy	131	73.6	114	77.6	245	75.4
Somewhat easy	46	25.8	27	18.4	73	22.5
Somewhat difficult	1	0.8	5	3.4	6	1.8
Very difficult	0	0	1	0.7	1	0.3
Primary reason for frequenting the food distribution site that day ^a^			149		149	
I obtain food at this site	------	------	98	65.8	98	65.8
A family member who obtains food/services at this site told me about the screening	------	------	18	12.1	18	12.1
A friend who obtains food/ services at this site told me about the screening	------	------	13	8.7	13	8.7
Other reason ^b^	------	------	20	13.4	20	13.4

^a^ This question was added for the Sallisaw health screenings after project staff observed that not all individuals at the Tahlequah screenings were attending to obtain vouchers for food to gain a better understanding of the sample population

^b^ Other reasons included working/volunteering at the site, obtaining other services at the site, supporting others with obtaining food/services at the site

### HCV Screening Results

92% (92.1%, n = 313) pilot participants received screening for HCV. Among these individuals, 167 (53.4%) participated in screenings at the Tahlequah site and 146 (46.6%) participated in screenings from the Sallisaw site. Eleven (3.5%) individuals received positive HCV antibody results across the two sites. Among the 11 individuals with positive HCV antibody results, five did not have a recorded history of HCV (Tables 4 and 5).

**Table 4:** Health screening results among 340 food distribution site project participants, 2019

Characteristic	Tahlequah (n = 184)		Sallisaw (n = 156)		Total (N = 340)	
	n	%	n	%	N	%
Blood pressure	181		77		258	
High ( > = 140/90)	83	45.9	32	41.6	115	44.6
Normal (90/60–140/90)	98	54.1	45	58.4	143	55.4
Body Mass Index (BMI)	198		12		210	
Obese	111	56.1	6	50.0	117	55.7
Overweight	50	25.3	5	41.7	55	26.2
Normal weight	34	17.2	0	0.0	34	16.2
Underweight	3	1.5	1	8.3	4	1.9
Diabetes (A1C)	114		67		181	
High (> 6.0)	35	30.7	25	37.3	60	33.1
Normal (4.5-6.0)	79	69.3	41	61.2	120	66.3
Low (< 4.5)	0	0	1	1.5	1	0.6
Diabetes (eAG)	111		0		111	
High (> 126 mg/dl)	34	30.6	------	------	34	30.6
Normal (70–126 mg/dl)	76	68.5	------	------	76	68.5
Low (< 70 mg/dl)	1	0.9	------	------	1	0.9
Cholesterol	115		73		188	
High (> 200)	30	26.1	16	21.9	46	24.5
Normal ( < = 200)	85	73.9	57	78.1	142	75.5
HDL	114		0		114	
High (> 60)	7	6.1	------	------	7	6.1
Normal (40–60)	52	45.6	------	------	52	45.6
Low (< 40)	55	48.2	------	------	55	48.2
LDL	114		0		114	
High (> 100)	67	58.8	------	------	67	58.8
Normal (0-100)	47	41.2	------	------	47	41.2
Triglycerides	114		0		114	
High (> 150)	61	53.5	------	------	61	53.5
Normal ( < = 150)	53	46.5	------	------	53	46.5
HCV ^a^	167		146		313	
Negative	164	98.2	138	94.5	302	96.5
Positive	3	1.8	8	5.5	11	3.5
HIV	112		78		190	
Negative	112	100	77	98.7	189	99.5
Positive	0	0	1	1.3	1	0.5
Syphilis (RPR)	115		79		194	
Negative	114	99.1	79	100	193	99.5
Positive	1	0.9	0	0	1	0.5

^a^ Among the 11 individuals who received positive antibody test results for HCV, five had undetectable viral loads at follow-up and did not require treatment, while six had detectable viral loads. Among the six individuals with detectable viral loads, four initiated HCV treatment and two did not initiate treatment and were recorded as lost to follow-up. Among the four individuals who initiated treatment, two completed treatment. Among the 11 individuals with positive HCV antibody results, five had a history of injecting drugs, and seven each had experienced incarceration and homelessness in the past. Five of the 11 individuals did not have a previous history of HCV. Five of the six individuals who required HCV treatment were male

**Table 5:** Health history among 340 food distribution site project participants, 2019^a^

Characteristic	Tahlequah (n = 184)		Sallisaw (n = 156)		Total (N = 340)	
	n	%	n	%	N	%
History of Diabetes	96		153		249	
No	57	59.4	125	81.7	182	73.1
Yes	39	40.6	28	18.3	67	26.9
Diabetes addressed? (yes) ^b^	38	97.4	27	96.4	65	97.0
Diabetes medication, past 12 months (yes) ^b^	35	89.7	25	89.3	60	92.3
History of high cholesterol	97		153		250	
No	67	69.1	145	94.8	212	84.8
Yes	30	30.9	8	5.2	38	15.2
Cholesterol addressed? (yes) ^c^	29	96.7	7	87.5	36	94.7
Cholesterol medication, past 12 months (yes) ^c^	26	86.7	7	87.5	33	86.8
History of HCV	84		151		235	
No	82	97.6	146	96.7	228	97.0
Yes ^d^	2	2.4	5	3.3	7	3.0
HCV addressed? (yes) ^e^	2	100	4	80.0	6	85.7
HCV medication, past 12 months (yes)	0	0	4	80.0	4	57.1
History of HIV	80		152		232	
No	80	100	151	97.3	231	99.6
Yes	0	0	1	0.7	1	0.4
HIV addressed? (yes) ^f^	------	------	1	100	1	100
HIV medication, past 12 months (yes) ^f^	------	------	1	100	1	100
History of syphilis (no)	81	100	152	100	233	100

^a^ Health history is only included for those individuals who were previously enrolled in CNHS and thus had a recorded health history through CNHS

^b^ Percent taken from the number who have a history of diabetes

^c^ Percent taken from the number who have a history of high cholesterol

^d^ Those with a history of HCV were not the same individuals who tested positive for HCV during these screening

^e^ Percent taken from the number who have a history of HCV, not among total who screened positive

^f^ Percent taken from the number who have a history of HIV

**Fig. 2:**
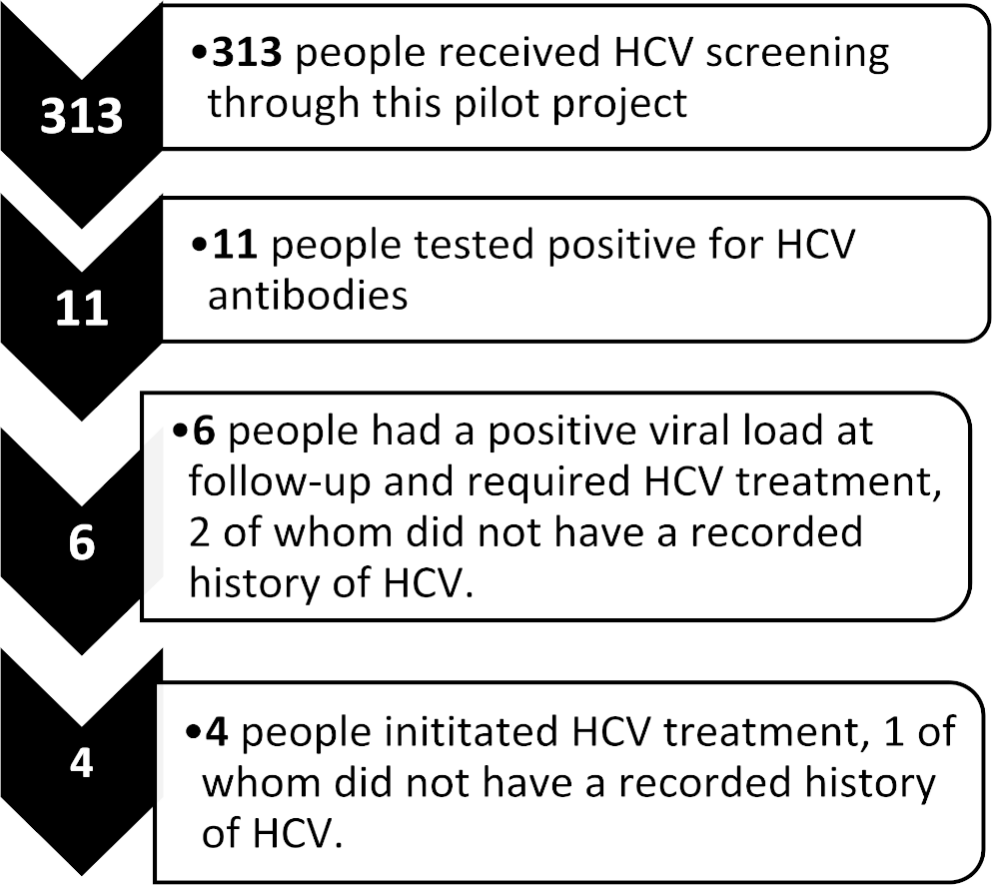
HCV screening, positivity, and treatment in Cherokee Nation as a result of pilot food distribution site screening, 2019

Of the 11 HCV seropositive individuals, six (54.5%) were found to have current HCV infection by quantifiable HCV RNA test, two (18.2%) of whom did not have a recorded history of HCV and four (67%) of whom initiated HCV treatment. Among the four individuals who initiated treatment, two completed treatment, while two were lost to follow-up after treatment initiation (Fig. 2**)**. In addition, five of the 11 individuals reported a history of injecting drugs, seven individuals had experienced incarceration in the past, and seven individuals had experienced homelessness in the past.

Demographic characteristics among those who were screened for HCV paralleled the demographic characteristics of those who received any health screenings at the food distribution sites (Table 6).

**Table 6:** Subset of demographics among 313 participants screened for HCV at food distribution sites, 2019

Characteristic	Total (N = 313)	
	N	%
Gender	312	
Female	203	65.1
Male	109	34.9
Age	310	
20 to 29 years	36	11.6
30 to 39 years	48	15.5
40 to 49 years	63	20.3
50 to 59 years	61	19.7
60 to 69 years	52	16.8
70 years or older	50	16.2
Marital status	312	
Single	135	43.3
Married	106	34.0
Divorced	41	13.1
Widowed	30	9.6
Education level	304	
High school or less	229	75.3
College (Associate level)	44	14.5
College (Bachelor level)	20	6.6
Graduate school	11	3.6
Employment status	308	
Not employed	201	65.3
Employed, full time	87	28.2
Employed, part time	20	6.5
Housing status	303	
Permanent housing	242	79.9
Temporary housing	51	16.8
Homeless	10	3.3
Insurance status	305	
Insured	165	54.1
No insurance	140	45.9
Incarceration, ever	303	
No	250	82.5
Yes	53	17.5

### Other Health Screening Results

Nearly half (44.6%) of participants exhibited high blood pressure ( > = 140/90) and more than half (55.7%, n = 117) of participants exhibited a BMI that put them in the obese range. Approximately one-third (33.1%, n = 60) of participants had high A1C (> 6.0) screenings, 24 (40.0%) of whom did not have a recorded history of pre-diabetes or diabetes. In addition, nearly one-quarter (24.5%) exhibited high (> 200) cholesterol screenings, 34 (73.9%) of whom did not have a recorded history of high cholesterol. No new cases of HIV were identified and only one new case of syphilis was identified through these health screenings (Tables 4 and 5**).**

## Discussion

This pilot project demonstrated that conducting HCV and other preventive health screenings at food distribution sites in Cherokee Nation is both feasible and acceptable to community members. There was high participation in all health screenings at the two food distribution sites in this pilot project. Most (76%) participants reported being very comfortable receiving health screenings at food distribution sites and that the screening process was very easy (75.4%).

A primary project goal was to reach community members that may not have easy access to health care. Conducting health screenings at food distribution sites allowed health workers to meet community members where they were. This potentially removed additional barriers to healthcare access, such as transportation, money or other logistical challenges, which may have prevented participants from receiving health care in traditional clinical settings. The high proportions of participants who had experienced homelessness, incarceration, and did not have a PCP demonstrated that conducting HCV and other health screenings at food distribution sites provided an opportunity to engage with individuals who may be facing substantial social and economic hardships. At their request, participants who were not currently enrolled in health services were input into the CNHS system and linked to care if they chose to seek additional health treatment and follow-up.

To reduce potential stigma associated with receiving HCV screening, HCV testing was offered as one of many healthcare screenings that participants could receive at the food distribution sites to support their long-term and short-term health. This pilot project demonstrated that offering health screenings at food distribution sites is an effective strategy for reaching and increasing the population of Cherokee Nation tribal citizens who are screened for HCV, in addition to other important chronic health conditions in Cherokee Nation. Through this project, clinical staff screened 313 individuals for HCV and identified six (1.9%) individuals for HCV treatment, two of whom may not have otherwise been identified as they did not have a recorded history of HCV. Individuals who frequented pilot food distribution sites exhibited a similar overall prevalence of HCV (1.9%) compared to the most recently reported HCV prevalence across Oklahoma (2.0%), with the prevalence among males (4.6%) slightly higher and females (0.5%) slightly lower in our pilot population compared with the HCV prevalence in Oklahoma (2.7% among males; 1.3% among females (30).

To our knowledge, there are no comparable community-based HCV screening programs that have been implemented in Indian Country. Two prior studies by Norton et al. and Kempf et al. similarly concluded that HCV screening is acceptable [39, 40] and feasible [40] in community settings. Norton et al. assessed acceptability across two homeless shelters, two drug rehabilitation centers, and one women’s drop-in center in Raleigh, North Carolina [39]. Kempf et al. assessed acceptability and feasibility among communities in rural Alabama and Mississippi [40]. Findings across urban community-based HCV screening programs, which included programs based in correctional facilities [16, 17, 21], mobile medical clinics [19, 20], community health centers and/or sexually transmitted infection clinics [17, 23], substance use disorder and/or syringe exchange programs [17, 23, 41], homeless shelters [17, 18], a health fair [42], a community pharmacy [25], and senior centers [24] varied widely, with HCV positivity ranging from 1.2% in a community pharmacy in San Francisco [25] to 86% among people living in homeless shelters in Los Angeles [18].

The findings in this study are subject to at least five limitations. First, since this pilot project was only implemented in two of the seven food distribution sites that Cherokee Nation operates, findings cannot be generalized to the full population of individuals who frequent food distribution sites across Cherokee communities. Second, there was heavy rain and flooding that occurred during the weeks that screenings were offered in Sallisaw, which may have prevented certain groups of individuals from frequenting the site who otherwise would have, leading to selection bias. Third, participants who frequented the sites during the later health screening days may also represent a biased sample. Approximately one-fifth (20.8%) of participants reported going to the food distribution site specifically for the health screening upon recommendation from a friend or family member. In this way, participants may be more similar to each other than they would be in a random sample of individuals who frequent food distribution sites. Fourth, providing participants with a gift card may have influenced recruitment for this pilot project, incentivizing individuals to obtain health screenings who otherwise may not have participated. Fifth, although insurance, employment, and housing status were collected, neither income status nor information on those who declined to participate in the health screenings were collected, which may have provided a broader understanding of the participant sample.

Future programs are needed to scale-up health screenings across food distribution sites, as well as to identify additional locations to engage community members in health screening within Cherokee Nation. Through identifying alternative HCV screening approaches, project staff successfully increased the number of individuals screened and treated for HCV in Cherokee Nation, improving equity in HCV related care. Through identifying and learning additional strategies to screen individuals in Cherokee Nation for HCV who otherwise may have been missed, and through engaging new Cherokee individuals in CNHS, this project supported overall health and HCV elimination efforts in Cherokee Nation.

## Conclusion

Limited research on strategies to engage individuals in non-clinic-based screening have been conducted among AI/AN communities in the U.S. Findings from this project demonstrate that more strategies are needed to reach populations who are missed through current screening efforts, and that implementing preventive health screening at food distribution sites or similar community service-based sites may be a helpful strategy for engaging community members in healthcare, not only for HCV, but for other infectious and chronic health conditions. Results from this pilot project may be useful to other AI/AN communities hoping to expand non-clinic-based health screening programs.

## Data Availability

Data supporting the findings of this study are not publicly available to protect the privacy and confidentiality of participants. Please contact the corresponding author for additional information.
